# Pathogen Discovery

**DOI:** 10.1371/journal.ppat.1000002

**Published:** 2008-04-25

**Authors:** W. Ian Lipkin

**Affiliations:** Center for Infection and Immunity, Mailman School of Public Health of Columbia University, New York, New York, United States of America; The Scripps Research Institute, United States of America

“What has become clear to you since we last met?”

—Benjamin Franklin

Discovery—literally, the act of uncovering—implies a new and fundamental observation that changes the way in which we view and respond to the world (Image 1). Eureka (“I have found it”) moments are rare in science. Given the physiques of many scientists, this may be a good thing. Legend has it that when Archimedes discovered while bathing that the volume of an object could be calculated by finding the volume of water it displaced, he leaped out of the bathtub and ran naked through the streets of Syracuse proclaiming his discovery.

The rate of discovery of new microbes, and of new associations of microbes with health and disease, has accelerated over the past two decades. Many factors are implicated. New pathogens have truly emerged with the globalization of travel and trade, changes in demographics and land use, susceptibility to opportunistic organisms associated with immunosuppression, and climate change [1]. New molecular technologies such as MassTag PCR [2]–[5], microbial microarrays [6]–[9], and unbiased high throughput sequencing (HTS) [10] have enabled efficient microbial surveillance and discovery. The databases needed to recognize sequences as host or microbial have improved dramatically. Sample collection has become sophisticated and comprehensive. Last, but not least, our models for pathogenesis embrace increasingly complex mechanisms that consider host–microbe–timing interactions in acute and chronic disease.

**Image 1:**
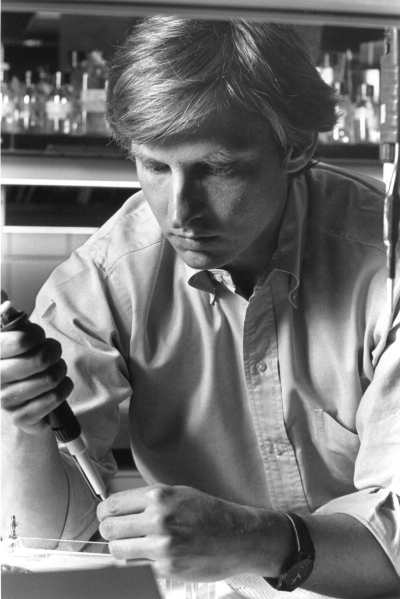
The author engaging in the process of scientific discovery.

## Proof of Causation

Finding an organism is only one step in establishing a causal relationship or understanding how it causes disease. Many have wrestled with the challenge of codifying the process of proving causation. Based on the germ theory of disease of Pasteur, Koch and Loeffler proposed criteria that define a causative relationship between agent and disease: the agent is present in every case of a disease; it is specific for that disease; and it can be propagated in culture and inoculated into a naïve host to cause the same disease [11]. Known as Koch's postulates, these criteria were modified by Rivers for viruses [12], and by Fredericks and Relman to reflect the introduction of molecular methods [13]. Although fulfillment of Koch's postulates remains the most persuasive evidence of causation, there are problems with holding to this standard. Overlap in signs and symptoms due to infection with different agents is common. Results of infection may vary with genetic background, age, nutrition, and previous exposure to similar agents. Many agents cannot be cultured; furthermore, there may be no animal model. Proving causation is particularly challenging where agents have remote effects or require co-factors for expression. In many acute infectious diseases, the responsible agent is readily implicated because it replicates at high levels in the affected tissue at the time the disease is manifest, morphological changes consistent with infection are evident, the agent is readily identified with classical or molecular methods, and there is evidence of an adaptive immune response. However, implication is more difficult when classical hallmarks of infection are absent or mechanisms of pathogenesis are indirect or subtle. Here, one may resort to a statistical assessment of the strength of epidemiological association based on the presence of the agent or its footprints (nucleic acid, antigen, and preferably, an immune response), and biological plausibility as indicated by analogy to diseases with other organisms where linkage is persuasive.

## Pathways to Pathogenesis

Implication of an agent is easiest if it is present in high concentration at the site of disease when the disease is manifest. Examples include poliomyelitis, where death of infected motor neurons results in paralysis, or infectious diarrheas where the causative agent (bacterium, virus, or parasite) is found in the gastrointestinal tract. More complex examples of intoxication occur in botulism or tetanus, where replication in the subcutaneous tissues or the gastrointestinal tract results in release of toxins that have remote effects on the nervous system. Pathogenesis may be immune-mediated as in hepatocellular carcinoma due to persistent hepatitis B or hepatitis C infections [14],[15]. Infection can also impair immune function, opening the door to opportunistic pathogens. This observation dates back to the early 1900s when von Pirquet noted the loss of skin reactivity to tuberculin in association with measles infection [16]; however, it is now best known in the context of HIV/AIDS. The effects of infection may depend on the age and maturation status of the host. Individuals at either extreme of life are at increased risk for morbidity and mortality [17]–[19]. Infection during organogenesis may have different consequences than at other times. Birth defects can accompany prenatal infection with toxoplasma, rubella-, cytomegalo-, and herpesviruses [20]. Persistent viral infections can have subtle effects on cellular physiology that result in alterations in the expression of neurotransmitters or hormones that have profound effects, including cognitive impairment [21], hypothyroidism [22], or diabetes mellitus [23]. Infection can break tolerance for “self,” resulting in autoimmune disease [24]. Autoimmunity may be restricted to the tissue in which the agent replicates, presumably because host antigens in that tissue are presented in a new context. However, cross-reactive immune responses may have an impact at a distance as in molecular mimicry, where streptococcal infection in the pharynx or the skin results in immunity to the heart and brain, causing valvular disease and chorea, respectively [25],[26]. The constellation of direct and indirect pathways to disease, short and long term, poses challenges for pathogen discovery.

## Strategies for Pathogen Discovery

Although reviews on pathogen surveillance and discovery typically focus on the latest molecular technologies, it is important to emphasize the pivotal roles of clinical acumen, pathology, serology, and classical culture techniques. Clinicians and epidemiologists are the prime movers in pathogen discovery. They recognize the appearance of new syndromes, collect materials for investigation, and persuade their colleagues to take up the search. Anatomic pathology can be instrumental in directing molecular work. The discoveries of Nipah virus [27] and West Nile virus [28]–[31] were facilitated by demonstration of henipavirus and flavivirus proteins in tissues, which allowed focused consensus PCR analyses. Classical virological methods such as tissue culture and serology proved pivotal in the 2003 SARS outbreak [32]. Propagation of a virus in tissue culture enabled its rapid characterization by consensus and random PCR cloning, microarray, and electron microscopy.

The advent of methods for cloning nucleic acids of microbial pathogens directly from clinical specimens ushered in a new era in pathogen discovery. Over the past two decades, subtractive cloning, expression cloning, consensus PCR, and high throughput pyrosequencing resulted in identification of novel agents associated with both acute and chronic diseases, including Borna disease virus, hepatitis C virus, Sin Nombre virus, HHV-6, HHV-8, *Bartonella henselae*, *Tropheryma whippelii*, Nipah virus, SARS coronavirus, and Israel Acute Paralysis virus [10], [27], [32]–[40].

The most familiar molecular assays in clinical microbiology are singleplex PCR assays designed to detect and quantitate the burden of individual candidate organisms. These have revolutionized blood banking, drug management of HIV, and containment of outbreaks of infectious disease. Degenerate primers can be employed in singleplex PCR assays at reduced stringency to facilitate detection of related but unknown organisms. However, clinical manifestations are not typically pathognomonic of infection with specific pathogens; thus, unless an investigator has clues to narrow a search, this is a cumbersome strategy even if samples, resources, and time are sufficient to invest in many singleplex assays for different agents.

In contrast, multiplex assays simultaneously entertain many hypotheses. The number of candidates considered can range from less than ten with multiplex PCR, to thousands with microarrays, to the entire tree of life with HTS. Costs and ease of use are improving; nonetheless, only multiplex PCR assays are widely used at present. In microarrays and HTS, many genetic targets compete for assay components (e.g., nucleotides, polymerases, and dyes), in some instances with different efficiencies. This abrogates quantitation and reduces sensitivity.

## A Staged Strategy for Pathogen Detection and Discovery

Costs of pathogen discovery can be contained by staging investment. Where epidemiology, serology, or pathology suggest one or a few candidates, singleplex PCR is ideal. Where no such clues pertain or singleplex assays fail, syndromic multiplex PCR assays allow rapid examination of up to 30 candidates with only a modest increase in time and expense. Microarrays are indicated if multiplex PCR fails. Each of these methods requires that an agent be related to one already known. Agents novel or sufficiently distant in sequence to confound hybridization may require subtractive cloning or HTS. Irrespective of the route that results in identification of a candidate, subsequent steps include quantitation of pathogen burden in affected hosts and controls, detailed characterization of the pathogen for features that may contribute to virulence or provide clues to provenance, and serology as an index to acute infection and as a tool to examine prevalence of infection over time and geography.

## Future Perspectives

Nucleic acid platforms are continually evolving, enabling faster, more sensitive, and less expensive methods for direct microbial detection. Improvements in development for these platforms include microfluidic sample processing and direct measurement of conductance changes with hybridization. Proteomics and host response profiling may yet yield diagnostic biomarkers. The most advanced technology will fail if samples are degraded, and data will be uninterpretable without accurate information on clinical course and sample provenance; thus, emphasis should be placed on engaging clinicians as equal partners. In chronic diseases, early exposure and/or genetic susceptibility may influence pathogenesis. The most substantive advances in linking microbes to disease may come from investments in prospective serial sample collections and models wherein diseases reflect intersections of genes and environment in a temporal context.
